# The Bidirectional Relationship Between FGIDs and Anxiety: Pathophysiological Mechanisms and New Therapeutic Strategies

**DOI:** 10.62641/aep.v53i4.1852

**Published:** 2025-08-05

**Authors:** Zhaoxia Liu

**Affiliations:** ^1^Gastroenterology Department, First Affiliated Hospital, Heilongjiang University of Chinese Medicine, 150001 Harbin, Heilongjiang, China

**Keywords:** functional gastrointestinal disorders (FGIDs), anxiety disorders, brain-gut axis, bidirectional relationship

## Abstract

Functional gastrointestinal disorders (FGIDs) encompass a group of disorders characterized by chronic or recurrent gastrointestinal symptoms, while anxiety disorders comprise a class of mental disorders primarily characterized by excessive anxiety and fear. Comorbidity of FGIDs and anxiety disorders has been frequently observed in clinical practice; however, the complex bidirectional relationship between these two disorders remains poorly understood. This review aimed to explore the bidirectional relationship between FGIDs and anxiety disorders, elucidate potential pathophysiological mechanisms, and propose novel diagnostic and therapeutic strategies. Through a review of recent literature, significant reciprocal factors that influence these two disorder categories have been identified. The prevalence of anxiety disorders among FGID patients is substantially higher than that in the general population; additionally, FGID symptoms are more prevalent in individuals with anxiety disorders. The core mechanisms underlying this bidirectional relationship likely involve dysfunction of the brain-gut axis, resulting from nervous, endocrine, and immune system dysfunction. Furthermore, intestinal dysbiosis, genetic factors, and early life stress may play crucial roles in this process. In terms of therapeutic strategies, innovative interventions for the effective management of comorbid FGIDs and anxiety disorders are proposed. Specifically, pharmacological interventions, including the use of selective 5-hydroxytryptamine (5-HT3) receptor antagonists and antidepressants, such as selective serotonin reuptake inhibitors (SSRIs) and serotonin-norepinephrine reuptake inhibitors (SNRIs), can alleviate both gastrointestinal and anxiety symptoms, while psychological interventions, such as cognitive behavioral therapy (CBT) and mindfulness-based stress reduction (MBSR), have also shown efficacy in the reduction of anxiety while significantly improving FGID symptoms. Furthermore, modulation of the gut microbiota through various interventions, such as administration of probiotics and low-Fermentable Oligo-, Di-, Mono-saccharides And Polyols (FODMAP) diets, has also been highlighted as a promising direction for future treatment. Given the collected evidence, the most effective approach would most likely be an integrated therapeutic model, which combines pharmacological, psychological, microbiota modulation, and lifestyle management through a multidisciplinary approach, all of which aim to deliver personalized, comprehensive treatment plans. In summary, the current review elucidates the bidirectional relationship, pathophysiological mechanisms, and novel therapeutic strategies for the treatment of comorbid FGIDs and anxiety disorders, proposing an integrative diagnostic approach that emphasizes screening for anxiety disorders in FGID patients and assessing gastrointestinal symptoms in patients with anxiety disorders. This comprehensive review aimed to provide a theoretical foundation for clinical practice and illuminate directions for future research, ultimately seeking to improve diagnostic and treatment outcomes and quality of life enhancement for this population.

## Introduction

Functional gastrointestinal disorders (FGIDs) include a range of chronic gastrointestinal symptoms without any identifiable organic pathology [1]. These disorders significantly affect the quality of life and place a substantial burden on healthcare systems [2]. Further, anxiety continues to be one of the most common mental disorders worldwide, with global lifetime prevalence continuing to increase. For instance, from 1990 to 2019, the global disability adjusted life year (DALY) rates for anxiety and major depressive disorder due solely to bullying increased by 23.31% and 26.60%, respectively [3]. Recent evidence has shown a bidirectional relationship between FGIDs and anxiety disorders, with an epidemiological study showing a markedly higher incidence of anxiety disorders among patients with FGID compared to the general population, and vice versa [4]. This comorbidity not only intensifies symptom burden but also complicates treatment approaches and increases healthcare resource utilization [5].

A thorough understanding of the bidirectional relationship between FGIDs and anxiety disorders is essential for uncovering their underlying pathophysiological mechanisms, with the ultimate goal of enhancing clinical management strategies. Currently, the brain-gut axis theory serves as a central framework for explaining this bidirectional interaction [6], highlighting the complex interplay among the central nervous, enteric nervous, endocrine, and immune systems [7]. Additionally, research on the gut microbiome has provided new insights into this relationship. Studies suggest that gut microbiota may impact emotions and behavior through multiple pathways, while the psychological state of the host may in turn influence the microbiome composition [8]. Despite extensive research, however, many questions remain about the exact mechanisms driving FGID/anxiety disorder comorbidity.

Given the high prevalence and complexity of these comorbid disorders, the development of effective diagnostic and therapeutic strategies has become increasingly important. Traditionally, these two categories of disorders were managed separately; however, in recent years, integrated treatments have drawn significant attention [9]. This approach emphasizes multidisciplinary collaboration, combining expertise from gastroenterology, psychiatry, psychology, and other related fields to deliver comprehensive and individualized treatment plans [10]. The current review systematically examines recent research on the bidirectional relationship between FGIDs and anxiety disorders, explores potential pathophysiological mechanisms, and proposes novel diagnostic and therapeutic strategies, with the aim of providing a theoretical foundation for clinical practice and outlining directions for future research. The ultimate goal of the current study is to help improve diagnostic and treatment outcomes and enhance the quality of life for patients with comorbid FGIDs and anxiety disorders.

## FGIDs and Anxiety Disorders: Clinical Features

### Definition and Classification of FGIDs

**Fig. 1.**
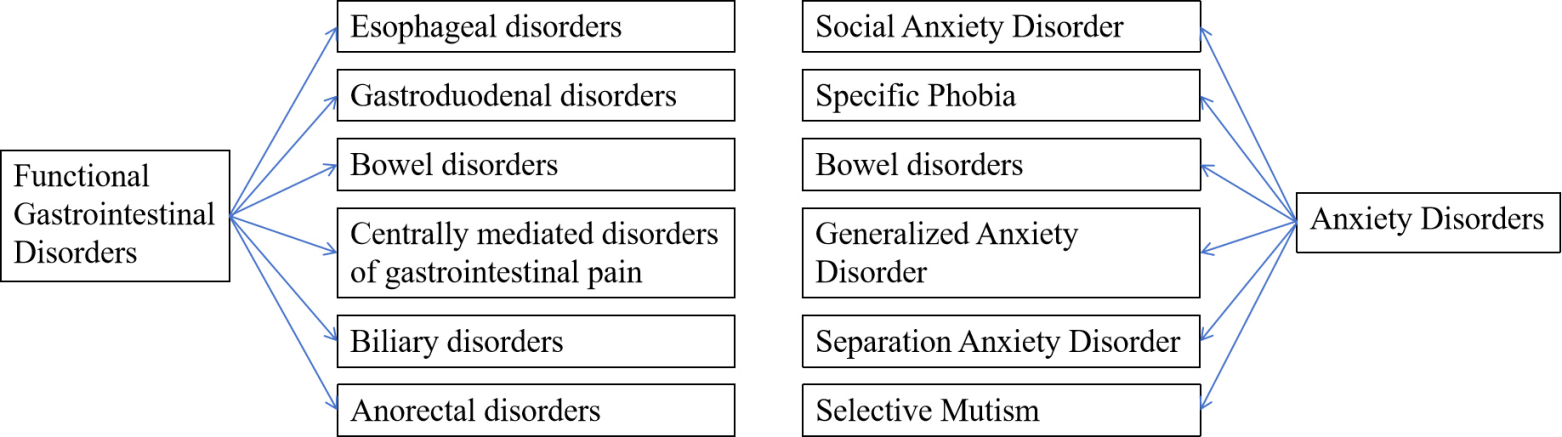
**Functional gastrointestinal disorders (FGIDs) and anxiety disorder classification map**. FGIDs into six main categories: esophageal disorders, gastroduodenal disorders, bowel disorders, centrally mediated disorders of gastrointestinal pain, biliary disorders, and anorectal disorders; Anxiety disorders encompass generalized anxiety disorder (GAD), panic disorder, specific phobia, social anxiety disorder, separation anxiety disorder, and selective mutism. This image is an original image, and its production software is WPS Office (12.1.0.16388, Kingsoft Corporation, Beijing, China).

According to the latest Rome criteria (Rome IV), FGIDs are classified as disorders of gut-brain interaction (DGBI) [11]. This classification emphasizes the crucial role of the bidirectional interactions observed between the central and enteric nervous systems in the development and progression of these disorders. Rome IV divides FGIDs into six main categories: esophageal disorders, gastroduodenal disorders, bowel disorders, centrally mediated disorders of gastrointestinal pain, biliary disorders, and anorectal disorders [12]. Among these, the most prevalent FGIDs include functional dyspepsia (FD) and irritable bowel syndrome (IBS). FD is primarily characterized by various symptoms, such as postprandial fullness, early satiety, epigastric pain, and/or burning sensation, while IBS is typically characterized by abdominal pain and changes in bowel habits [13] (Fig. 1).

### Definition and Classification of Anxiety Disorders

Anxiety disorders are a class of mental health conditions marked by excessive anxiety and fear. According to the Diagnostic and Statistical Manual of Mental Disorders, Fifth Edition (DSM-5) from the American Psychiatric Association, anxiety disorders include generalized anxiety disorder (GAD), panic disorder, specific phobia, social anxiety disorder, separation anxiety disorder, and selective mutism [14]. Among these, GAD is one of the most common forms, characterized by persistent and excessive worry that is difficult to control, along with various physical symptoms, such as muscle tension, fatigue, and difficulty concentrating [15]. Panic disorder, however, involves recurrent episodes of fear or discomfort, often accompanied by physiological symptoms, such as palpitations, sweating, trembling, and chest tightness [16]. Notably, patients with anxiety disorders frequently experience gastrointestinal symptoms, such as nausea, abdominal pain, and diarrhea [17] (Fig. 1).

### Comorbid Patterns and Clinical Presentations

Comorbidity of FGIDs and anxiety disorders has been frequently observed within clinical settings. Further, research has shown that the prevalence of anxiety disorders among patients with FGIDs is significantly higher than that observed in the general population; the reverse is also true [18]. For example, over one-third of patients with IBS exhibit symptoms of anxiety disorders [10], with a similar proportion of anxiety symptoms seen in patients with FD [19]. This pattern of comorbidity not only worsens symptom severity but can also impact disease prognosis and treatment efficacy. Clinically, patients with both conditions often exhibit more severe gastrointestinal symptoms and heightened anxiety. For instance, IBS patients with comorbid anxiety disorders may experience more frequent abdominal pain, altered bowel habits, and heightened anxiety [20]. Further, these patients often exhibit increased stress sensitivity and lower pain thresholds [21]. Notably, FGIDs and anxiety symptoms can exacerbate each other, creating a positive feedback loop [22]. Therefore, screening for anxiety symptoms in FGID patients and evaluating for gastrointestinal symptoms in individuals with anxiety disorders are crucial steps to increase early detection, thus improving effective management of this comorbidity.

## Epidemiological Evidence of Bidirectional Relationships

### Prevalence of Anxiety Disorders in Patients With FGIDs

Numerous studies have consistently demonstrated significantly higher prevalence of anxiety disorders among patients with FGIDs, compared to the general population. For example, a study involving 335 adults found a notable increase in the prevalence of GAD among patients with IBS [23]. Lee *et al*. [24] reported a 12-month prevalence of GAD of 4%, with an incidence rate five times higher in IBS patients than those without IBS (odds ratio (OR): 5.84, *p* < 0.001). Another study on FD showed that anxiety disorders were present in 26.98% of FD patients, significantly exceeding the 18.9% typically seen in healthy controls [25]. Evidence suggests that incidence rates of anxiety disorders also vary widely across different types of FGIDs. For instance, a study on functional abdominal pain syndrome (FAPS) found that up to 51% of FAPS patients had at least one psychiatric disorder, which was a proportion even higher than that observed in IBS patients [26]. These findings underscore the strong association between FGIDs and anxiety disorders and highlight the importance of screening and assessing anxiety symptoms in patients with FGIDs (Table 1, Ref. [23, 24, 25, 27]).

**Table 1.** **The bidirectional relationship between FGID and anxiety disorders**.

Baseline diseases (Incidence, %)	Comorbid conditions	References
IBS	The prevalence of GAD is significantly increased among patients with IBS	[23]
IBS (5.4%)	The incidence of IBS in GAD responders is 4.7 times that of non-GAD responders	[24]
FGID (34.7%)	The risk of developing IBS in patients with severe anxiety symptoms is 2.7 times that of patients with mild anxiety symptoms	[27]
GAD (4%)	The incidence of GAD in IBS responders is five times that of non-IBS responders	[24]
GAD (18.9%)	The incidence of anxiety disorders among FD patients is as high as 26.98%	[25]
FAPS	The incidence of at least one psychiatric disorder among FAPS patients is as high as 51%	[25]

IBS, irritable bowel syndrome; FGID, functional gastrointestinal disorder; GAD, generalized anxiety disorder; FAPS, functional abdominal pain syndrome; FD, functional dyspepsia.

### Prevalence of FGIDs in Patients With Anxiety Disorders

Incidence rates of FGIDs among individuals with anxiety disorders are also significantly higher than those observed in the general population. For instance, a cross-sectional study of 2005 participants found an overall IBS prevalence of 5.4%; however, IBS incidence among individuals with GAD was found to be 4.7 times higher than that in those without GAD (OR: 6.32, *p* < 0.001) [24]. Additionally, severity of anxiety symptoms positively correlates with the risk of developing FGIDs. A five-year longitudinal study showed that patients with severe anxiety symptoms exhibited a 2.7-fold greater risk of developing IBS than those with mild anxiety symptoms [27]. These findings suggest strong underlying mechanisms for both conditions and highlight the importance of assessing gastrointestinal symptoms in patients with anxiety disorders (Table 1).

### Longitudinal Studies

Longitudinal studies offer deeper insights into the bidirectional relationship between FGIDs and anxiety disorders. These studies not only reveal the temporal sequence of the two conditions but also suggest potential causal links between them. Research has shown that individuals with FGIDs at baseline have a 2.2-fold higher risk of developing anxiety disorders during follow-up [28]. Additionally, anxiety symptoms have been identified as independent predictors for the development of IBS [27], while IBS symptoms are similarly associated with a higher risk for the later onset of anxiety disorders [27]. A 10-year follow-up study of individuals with FD found that baseline anxiety symptoms were associated with a 3.1-fold greater risk of developing FD compared to those without anxiety [29]; this study also revealed a positive correlation between FD symptom duration and anxiety severity [29]. These longitudinal studies confirm the bidirectional relationship between FGIDs and anxiety disorders, providing valuable temporal information insights into the developmental trajectories and mutual influences of these two conditions.

## Potential Pathophysiological Mechanisms

### Brain-Gut Axis Theory

**Fig. 2.**
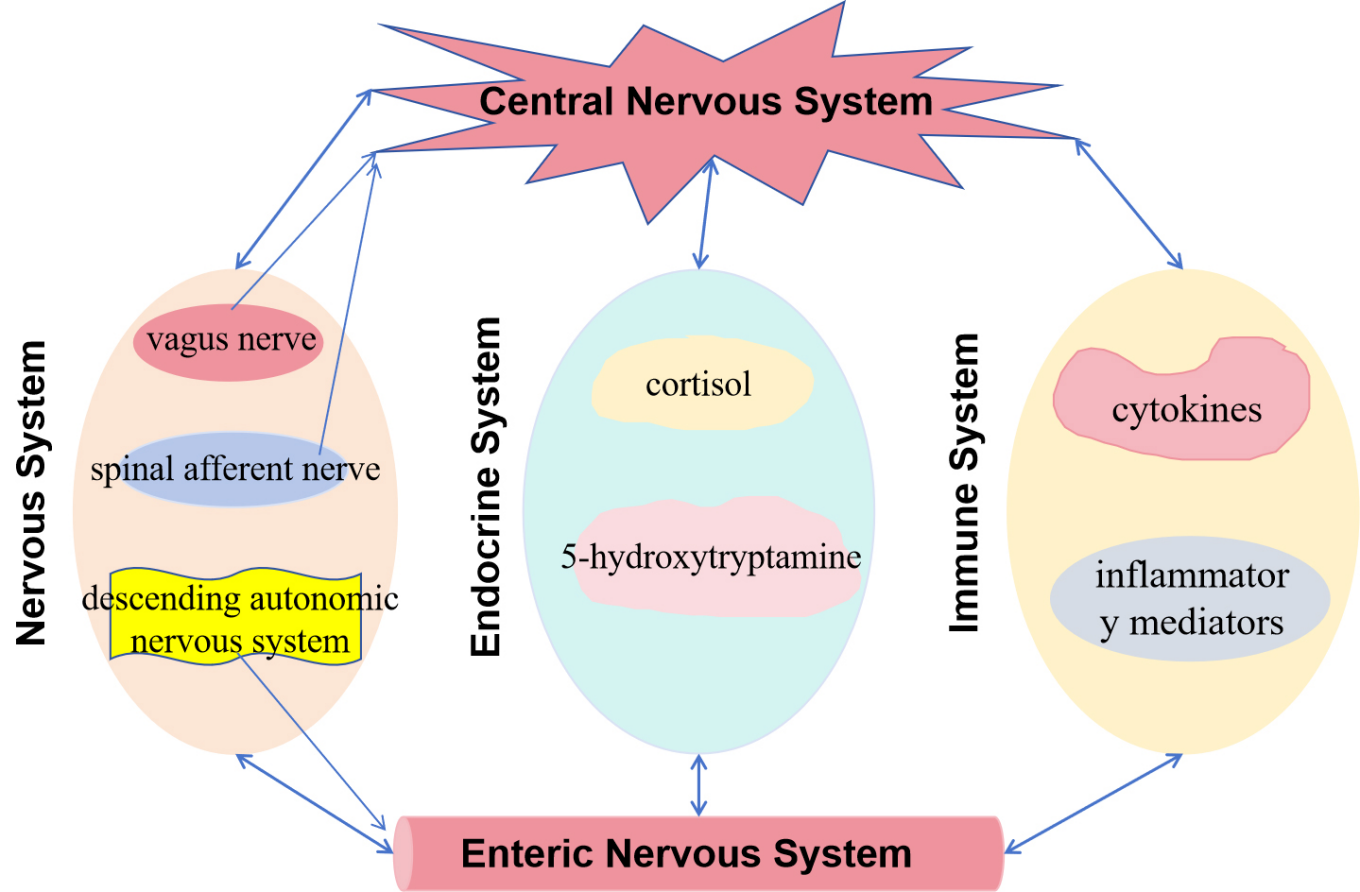
**Mechanism diagram of brain-gut axis theory**. This image is an original image, and its production software is WPS Office (12.1.0.16388, Kingsoft Corporation, Beijing, China).

The brain-gut axis theory is a key concept in explaining the pathogenesis of FGIDs. Specifically, this theory highlights the critical role of bidirectional interactions between the central and enteric nervous systems in FGID development [30]. The brain-gut axis communicates through multiple routes, including neurological, endocrine, immune, and humoral pathways. In the nervous system, the vagus and spinal afferent nerves transmit sensory information from the gut to the brain, while the descending autonomic nervous system regulates intestinal function. The endocrine system is influenced by several different hormones, while the immune system affects gut function and sensation via cytokines and inflammatory mediators (shown in Fig. 2). This complex interaction allows emotional states, stress, and cognitive processes to impact gastrointestinal function and vice versa [31]. For example, studies have demonstrated that patients with FGIDs often exhibit brain-gut axis dysfunction, such as visceral hypersensitivity, intestinal motility disorders, and abnormal central processing of visceral information [32]. Thus, the brain-gut axis theory provides a comprehensive framework to better understand the diverse symptoms and complex pathophysiological mechanisms of FGIDs and suggests directions for developing new therapeutic strategies.

### Dysregulation of Intestinal Flora

Intestinal flora disorder plays an increasingly significant role in the pathogenesis of FGIDs. The human gut hosts trillions of microbes that form complex symbiotic relationships with their hosts. These microorganisms are involved in food digestion and absorption, as well as in immune regulation, maintaining intestinal barrier function, and influencing the central nervous system [33]. Recent studies have even shown that patients with FGIDs will often exhibit abnormalities in gut microbiota composition and function. For instance, IBS patients frequently show a decrease in beneficial bacteria, such as lactobacillus and bifidobacterium, with a corresponding increase in potentially pathogenic bacteria, such as Bacteroides [34]. This microbial imbalance can lead to various pathophysiological changes, including increased intestinal permeability, localized inflammation, altered gut motility, and visceral hypersensitivity [13]. Furthermore, gut flora either directly or indirectly influence the gut-brain axis through the production of various metabolites, such as short-chain fatty acids and neurotransmitter precursors [35]. Factors such as diet, antibiotic use, and stress contribute to changes in the gut microbiota, helping explain why these factors may trigger or worsen FGID symptoms. Consequently, correcting intestinal flora imbalance has become a key therapeutic target for FGID treatment, and the effectiveness of interventions like probiotics and prebiotics is actively being explored.

### Genetic Factors

Genetics also play a significant role in the pathogenesis of FGIDs. Although FGIDs are not typically classified as a genetic disorder, familial clustering suggests that genetic factors are almost certainly involved in their development. Research shows that maternal psychological factors may be associated with the occurrence of FGIDs in offspring [36]. With advancements in genomics, researchers have identified several candidate genes associated with FGIDs. These genes are primarily associated with the neurotransmitter system, inflammatory response, immune regulation, and intestinal barrier function. For example, polymorphisms in the serotonin transporter gene (*SLC6A4*) are linked to an increased risk of IBS development and symptom severity [37]. Variations in genes encoding intestinal epithelial tight junction proteins, such as Cadherin 1 (*CDH1*), have also been shown to be associated with a higher risk of developing an FGID [38]. Additionally, polymorphisms in stress-related genes, such as the corticotropin-releasing hormone receptor 1 (*CRHR1*) gene, have been reported to increase susceptibility to IBS [39]. It is important to note that genetic factors alone are likely not responsible for FGID development, as these factors typically interact with environmental influences. For instance, certain gene variants may increase susceptibility to early life stress or infection; this, in turn, raises the risk of developing FGIDs later in life. This gene-environment interaction complexity explains why FGIDs do not follow simple Mendelian inheritance patterns. In the future, large-scale genome-wide association studies may provide a more comprehensive understanding of the genetic basis of FGIDs, aiding in disease risk prediction and supporting the development of individualized treatment strategies.

### Early Life Stress and Trauma

The experience of stress and trauma early in development is considered a significant risk factor for the development of FGIDs. Epidemiological and clinical study has shown that adverse childhood experiences (ACEs), such as abuse, neglect, and family dysfunction, are strongly associated with an increased risk of FGIDs in adulthood [40]. The likely mechanisms that underlie this association are complex, involving long-term changes in not only the neuroendocrine system but also in immune function regulation and gut microbiota composition. Early life stress may lead to permanent alterations in the hypothalamic-pituitary-adrenal (HPA) axis, causing abnormal responses to future stress [41]. For example, a study has found that IBS patients who were abused in childhood exhibit elevated baseline cortisol levels and stress reactivity when compared to individuals who did not experience abuse [42]. In addition, early life stress may also affect gene expression through epigenetic modification, thereby altering stress sensitivity and coping [43]. At the nervous system level, early traumatic experiences may lead to alterations in central nervous system processing of visceral sensory information, increasing the risk of visceral hypersensitivity. Animal experiments have shown that neonatal stress can lead to increased intestinal permeability, low-grade inflammation, and increased visceral sensitivity into adulthood [44]. It is important to note that the effects of early life stress can be enduring, possibly lasting a lifetime. This “biological programming” explains why adverse childhood experiences can continue to impact gastrointestinal function well into later life. Thus, addressing the early life experiences of patients in an effort to provide targeted psychological support and intervention may be a key strategy to prevent and treat FGIDs.

### Chronic Stress and Dysfunction of the HPA Axis

Chronic stress and HPA axis dysfunction are critical to understanding the pathophysiological mechanisms that underlie FGIDs. In modern society, chronic stress has become increasingly common, resulting in continuous activation of the stress response system, specifically the HPA axis. The HPA axis is a neuroendocrine pathway that responds to stress, and its dysfunction is closely linked to the onset and exacerbation of various FGIDs [45]. Under normal conditions, the perception of stress by an organism triggers the hypothalamus to release corticotropin releasing factor (CRF), which stimulates the pituitary gland to secrete adrenocorticotropin hormone (ACTH), ultimately stimulating the adrenal cortex to release glucocorticoids (primarily cortisol). However, prolonged chronic stress can lead to dysregulation within this system, leading to altered cortisol secretion patterns, impaired negative feedback mechanisms, and overactivation of the CRF system [46]. These changes not only affect the functioning of the central nervous system but also act directly on the gut, leading to alterations in gastrointestinal function. For example, CRF has been shown to increase intestinal permeability, promote inflammation, and alter gut motility, all of which are common pathophysiological features of FGIDs [47]. Furthermore, HPA axis dysfunction can impact the composition and function of gut microbiota, further aggravating intestinal dysfunction [48]. Notably, patients with FGIDs often exhibit heightened stress reactivity, which may be partly due to HPA axis changes stemming from early life stress. This increased stress reactivity may exacerbate symptoms, creating a positive feedback loop. Therefore, in addition to the treatment of specific gastrointestinal symptoms, treatment of FGIDs should also address stress management and the regulation of HPA axis function. Some non-pharmacological approaches, such as cognitive behavioral therapy (CBT) and mindfulness-based stress reduction, have been shown to be effective in improving both stress levels and gastrointestinal symptoms in patients with FGIDs [49]. In the future, the development of targeted therapies focusing on the HPA axis may provide new treatment options for FGIDs.

## Diagnostic Strategy

### Diagnostic Methods of FGIDs and Application in Patients With Anxiety Disorders

The diagnosis of FGIDs and their occurrence in patients with anxiety disorder is a complex and important clinical issue. FGID diagnosis primarily relies on symptom-based criteria, with Rome IV clinical guidelines being the most widely utilized [50]. These criteria provide detailed diagnostic guidelines for various FGIDs, such as IBS, which requires abdominal pain persisting for at least 3 months and is associated with changes in bowel habits [51]. However, when applying these criteria to patients with anxiety disorders, special consideration is needed, as anxiety symptoms can influence how patients perceive and report gastrointestinal symptoms. For example, individuals with GAD may focus excessively on minor gastrointestinal discomfort, potentially leading to biased symptom reporting [28]. To assess FGIDs in such patients, in addition to standard symptom assessment, scales like the Functional Gastrointestinal Disease Quality of Life Scale (FGIDs-QOL) or the Gastrointestinal Symptom Rating Scale (GSRS) can help provide a more comprehensive evaluation of symptom impact [52, 53]. However, several tests, including routine blood work, stool analysis, and abdominal ultrasound, are often necessary to rule out organic diseases. In some cases, an endoscopy or other imaging may be required to further confirm the diagnosis [54]. It is important to interpret these test results with caution in patients with anxiety disorders, as anxiety can influence physiological indicators, such as intestinal permeability and markers of inflammation [55].

### Diagnosis of Anxiety Disorders and Application in Patients With FGIDs

The diagnosis of anxiety disorders in patients with FGIDs also requires special attention and primarily relies on clinical interviews and standardized diagnostic criteria, such as that found in the fifth edition of the DSM-5 or the 11th edition of the International Classification of Diseases (ICD-11) [56, 57]. However, applying these criteria to patients with FGIDs can present challenges. First, FGID symptoms themselves can induce anxiety, making it difficult to distinguish secondary and primary anxiety disorders [58]. Second, some gastrointestinal symptoms (such as nausea and abdominal pain) overlap with anxiety symptoms (such as tension and worry), complicating the diagnosis [59]. Therefore, when evaluating anxiety symptoms in patients with FGIDs, it is advisable to use tools specifically designed for this population, such as the Hospital Anxiety and Depression Scale (HADS) or the Generalized Anxiety Disorder-7 Scale (GAD-7) [60]. These tools can help differentiate between normal disease-related concerns and pathological anxiety. Structured clinical interviews, such as the Mini International Neuropsychiatric Interview (MINI) or the Structured Clinical Interview for DSM-5 (SCID), can also provide detailed diagnostic information [61]. During assessment, the timing and interaction between anxiety symptoms and gastrointestinal symptoms should be considered key pieces of information, all of which can be tracked through various methods, including symptom diaries or ecological momentary assessments (EMAs) [62]. It is worth noting that individuals with certain anxiety disorders (e.g., panic disorder) may exhibit significant gastrointestinal symptoms, making comprehensive psychiatric evaluation essential for patients with FGIDs.

### Proposal of Integrative Diagnostic Approaches

Given the complex interrelationship between FGIDs and anxiety disorders, integrative diagnostic approaches are particularly important. Such approaches should consider both gastrointestinal symptoms and psychological factors, employing a multi-dimensional and multi-disciplinary assessment strategy. For instance, biopsychosocial assessment models, such as the Multidimensional Clinical Profile (MDCP) developed by the Rome Work Group, can be utilized. The MDCP encompasses five dimensions: (1) clinical diagnosis, (2) physiological regulation dysfunction, (3) psychological and psychosocial factors, (4) daily function and quality of life impact, and (5) environmental influence factors. This model offers a comprehensive framework to better capture the complexity of comorbid FGIDs and anxiety disorders. Additionally, a staged assessment strategy can be implemented, beginning with initial screening (e.g., the Rome IV diagnostic questionnaire and the GAD-7 scale), followed by a more in-depth assessment based on screening results [63, 64]. Further, emerging technological tools (e.g., artificial intelligence-assisted symptom analysis systems) can be used to aid diagnosis, ultimately helping to identify complex symptom patterns and potential diagnoses [65]. Finally, given the dynamic nature of FGIDs and anxiety disorders, a longitudinal assessment strategy with periodic re-evaluation should be adopted to capture the natural progression of the conditions and the response to treatments [66].

## Novel Treatment Strategies

### Pharmacological Interventions

Pharmacological interventions play a crucial role in managing comorbid FGIDs and anxiety disorders. Historically, these conditions were treated separately; however, recent research suggests that certain medications may benefit both. For FGIDs, commonly prescribed medications include antispasmodics, laxatives, antidiarrheals, and prokinetics [67]; however, novel agents like linaclotide and prucalopride have also shown significant efficacy in treating IBS [68]. In treating anxiety disorders, selective serotonin reuptake inhibitors (SSRIs) and serotonin-norepinephrine reuptake inhibitors (SNRIs) are generally used as first-line interventions [69]. Interestingly, these antidepressants have also been found to improve certain FGID symptoms, particularly in reducing pain and abdominal discomfort [70]. For example, paroxetine has been shown to alleviate anxiety symptoms and reduce abdominal pain and diarrhea in IBS patients [71]. Additionally, emerging pharmacological agents, such as 5-hydroxytryptamine (5-HT3) receptor antagonists (e.g., ondansetron) and 5-hydroxytryptamine 4 (5-HT4) receptor agonists (e.g., tegaserod), may offer beneficial effects on both FGID symptoms and anxiety [72]. Recently, Gunn *et al*. [73] reported a clinical case demonstrating favorable outcomes in treating IBS with ondansetron. Additionally, Zhao *et al*.’s study [74] indicates that acupuncture, as an adjunct therapy to SSRIs, can improve treatment outcomes for anxiety-related depression, while Stasi *et al*.’s study [71] demonstrates significant improvement in both psychological and gastrointestinal symptoms in patients treated with paroxetine. However, it is crucial to emphasize that pharmacological management should be tailored to the individual, taking into account specific symptoms, comorbidities, and potential adverse effects.

### Psychological Interventions

Psychological interventions have become increasingly prominent in the management of comorbid FGIDs and anxiety disorders. Cognitive behavioral therapy is one of the most extensively studied and applied psychological treatment modalities, demonstrating efficacy in reducing anxiety symptoms while simultaneously enhancing patients’ ability to manage FGID symptoms [75]. CBT works by helping patients identify and modify maladaptive thought patterns and behaviors, thereby reducing the hypervigilance and catastrophizing that is often associated with gastrointestinal symptoms [5]. For instance, a study of patients with comorbid IBS and anxiety disorder reported significant improvements in both IBS symptoms and anxiety levels following a 12-week CBT intervention [76]. Mindfulness-based stress reduction (MBSR) has also shown promising results in this area. By enhancing patients’ bodily awareness and acceptance, MBSR helps reduce stress responses, which in turn alleviates both gastrointestinal symptoms and anxiety [77]. For example, a study of 47 IBS patients showed that, compared to the control group, IBS patients receiving MBSR for teens (MBSRT) demonstrated greater improvements in quality of life and mindfulness components, along with a reduction in IBS symptoms [78]. Gut-directed hypnotherapy is another emerging approach that has been shown to directly influence intestinal function through hypnotic techniques while simultaneously alleviating anxiety symptoms [79]. This method has shown particular effectiveness in treating refractory IBS cases [80]. Notably, a recent rise of Internet-based psychological interventions for FGIDs and anxiety disorders has been observed, which offer promising methods to improve treatment accessibility and adherence [81].

### Modulation of the Gut Microbiome

Modulation of the gut microbiome has emerged as a promising therapeutic strategy in managing comorbid FGIDs and anxiety disorders. As understanding of the gut-brain axis deepens, researchers have recognized the crucial role of intestinal microbiota in the pathogenesis and progression of both FGIDs and anxiety disorders [82]. Probiotics are one of the most widely used approaches to modulate the microbiome. A meta-analysis has shown that specific probiotic strains, such as Bifidobacterium and Lactobacillus species, can significantly alleviate IBS symptoms [83]. Intriguingly, study has suggested that probiotics may not only improve gastrointestinal symptoms but may also exert positive effects on anxiety symptoms [84]. For example, a prospective, multicenter, non-interventional study found that daily intake of a probiotic sachet (3 × 10^9^ Colony Forming Unit (CFU)) and vitamin D (10 µg) effectively alleviated symptoms of IBS, anxiety, and depression [85]. In addition to probiotics, prebiotics (e.g., fructo-oligosaccharides) and synbiotics (combinations of probiotics and prebiotics) also show therapeutic potential [86]. Fecal microbiota transplantation (FMT) is another emerging microbiome modulation approach. Although FMT is primarily used to treat refractory Clostridioides difficile infections, it has recently been explored as a treatment option for IBS and other FGIDs [87]. A small study suggested that FMT may improve gastrointestinal symptoms and quality of life in IBS patients [88], though its role in treating anxiety disorders requires further investigation. Dietary interventions, such as the low-Fermentable Oligo-, Di-, Mono-saccharides And Polyols (FODMAP) diet, are also important strategies for microbiome modulation; this diet has been shown to effectively reduce IBS symptoms and may positively impact psychological health [89].

### Integrative Treatment Model

The integrated treatment model has emerged as a promising strategy for the management of comorbid FGIDs and anxiety disorders, emphasizing multidisciplinary and multimodal interventions. This model addresses the complex interplay between FGIDs and anxiety disorders, aiming for optimal therapeutic outcomes by simultaneously targeting multiple pathophysiological mechanisms [90]. A typical integrated treatment protocol may include pharmacotherapy, psychological interventions, microbiome modulation, dietary management, and lifestyle modifications [58]. For example, a study of patients with comorbid IBS and anxiety disorder employed a comprehensive intervention protocol that combined SSRI medication, CBT, probiotic supplementation, and low-FODMAP dietary guidance, resulting in significant improvements in both gastrointestinal symptoms and anxiety levels [76]. Another key component of the integrated model is the stepped care approach, which gradually increases treatment intensity and complexity based on the severity of patient symptoms [91]. For example, patients with mild symptoms may begin with lifestyle modifications and self-management strategies, while those with severe or refractory symptoms may need more intensive pharmacological interventions and specialized psychological therapies [91]. Additionally, the integrated treatment model emphasizes patient education and self-management, as research has shown that empowering patients to understand and manage their conditions can enhance the long-term effectiveness of treatments [91]. Implementing an integrated treatment model requires close collaboration among a multidisciplinary team, including gastroenterologists, psychiatrists, psychotherapists, and nutritionists [91]. Although challenges exist, such as coordination difficulties and high costs, the complex and often refractory nature of comorbid FGIDs and anxiety disorders suggests that integrated treatment models may represent the future of management strategies.

## Limitations and Outlook

There are several limitations to consider when interpreting the current review. First, although a review of the bidirectional relationship and potential underlying mechanisms between FGIDs and anxiety disorders was conducted, some mechanistic inferences based on cross-sectional studies were made, making it difficult to establish causality. Additionally, many emerging therapeutic strategies require validation through larger-scale clinical trials to confirm their long-term efficacy and safety, thereby further supporting their clinical application. Looking ahead, research and management of these comorbid disorders are expected to increasingly emphasize multidisciplinary collaboration and a holistic approach. The integration of basic research within clinical practice will expedite the translation of laboratory findings into clinical applications. Further, big data and artificial intelligence technologies may revolutionize our understanding and management of these disorders through the identification of previously unknown associations. Nonetheless, several challenges remain. For instance, the clinical application of biomarkers requires extensive validation, the implementation of precision medicine models must consider cost-effectiveness and ethical concerns, and the long-term safety and efficacy of new therapeutic approaches have yet to be established. Additionally, integrating these advancements into existing healthcare systems, training medical personnel in new technologies, and ensuring equitable access to novel approaches are critical issues. However, as research progresses and technology advances, it is likely that the diagnosis and treatment of comorbid FGIDs and anxiety disorders will enter a new era, ultimately enhancing patients’ quality of life.

## Conclusion

This review thoroughly examined the complex bidirectional relationship between FGIDs and anxiety disorders, highlighting its significant implications for patient diagnosis, treatment, and prognosis. Through the analysis of interactions across multiple levels, including the brain-gut axis, neuroendocrine system, immune regulation, and genetic factors, the understanding of the shared pathophysiological mechanisms underlying these disorders has been deepened and a theoretical foundation for new therapeutic strategies has been provided. This comprehensive analysis emphasizes the importance of an integrated approach to managing comorbid FGIDs and anxiety disorders, potentially paving the way for more effective and personalized interventions in the future.

## Availability of Data and Materials

Not applicable.
